# YawnStim: a standardized and diverse video stimulus set

**DOI:** 10.7717/peerj.21434

**Published:** 2026-06-23

**Authors:** Omar Tonsi Eldakar, Brianna M. Calderón, Maya T. Totev, Jorg J.M. Massen, Andrew C. Gallup

**Affiliations:** 1 Biological Sciences, Nova Southeastern University, Fort Lauderdale, FL, United States of America; 2 Animal Behaviour and Cognition, Utrecht University, Utrecht, Netherlands; 3 Psychology, State University of New York (SUNY), Utica, NY, United States of America; 4 Psychological and Brain Sciences, The Johns Hopkins University, Baltimore, MD, United States of America

**Keywords:** Behavioral contagion, Facial mimicry, Motor resonance, Social cognition, Vigilance

## Abstract

In humans, contagious yawning is often elicited experimentally using a video stimulus, and individual differences in this response may be indicative of variation in important aspects of social cognition. However, researchers often use different and highly variable stimulus sets, making comparisons between studies and across conditions difficult. The lack of participant diversity within existing stimulus sets also limits the generalizability of previous findings. Here, a free stimulus set called YawnStim is described. YawnStim includes a series of standardized experimental and control videos from a diverse sample of men and women from the following self-identified categories: Caucasian, Black, Hispanic/Latino, and Asian. Videos are compiled into four conditions, including both (1) uncovered and (2) covered yawns, as well as (3) mouth closed and (4) mouth gaping controls. This stimulus package was validated in a series of five within-subjects laboratory experiments. Collectively, results from the individual experiments and accompanying mini meta-analyses demonstrated that both the uncovered and covered yawning conditions produced amplified self-reported yawning responses. Potential follow-up research both in terms of validation and further applications of YawnStim are discussed.

## Introduction

Contagious yawns are triggered or released automatically (or involuntarily) by the yawns of others (Provine, 1986). This type of response is a distinct form of facial mimicry and motor resonance shared by humans and highly social non-human animals (Palagi et al., 2020; Gallup, 2022), and is thought to result from a perception-action mechanism permitting the rapid synchronization of states between individuals (Preston & De Waal, 2002). The contagiousness of yawning is perhaps one of the most highly replicable effects within the field of psychology. Seeing, hearing, reading, or even thinking about others yawning can trigger this response (Provine, 2005), and we are not aware of a single study that has failed to elicit this effect. While various experimental stimuli have been employed to study yawn contagion (*e.g.*, Provine, 1986; Provine, 1989; Gallup & Eldakar, 2011; Helt et al., 2010; Massen, Church & Gallup, 2015; Gallup & Wozny, 2022), the most common method is to have participants watch videos of other people yawning (Platek et al., 2003). Indeed, a recent study comparing the responses of visual, auditory, and visual-audio yawning stimuli found that contagious yawning is driven primarily by the visual modality (De Weck et al., 2022).

One of the more striking features of contagious yawning is the variation in which individuals respond to yawning cues. Studies have consistently demonstrated substantial individual differences in contagious yawning, which is distinct from the primitive and universal form of this behavior, *i.e.,* spontaneous yawning (Gallup, 2011). Typically ∼30–70% of participants will yawn in response to visual stimuli presented in the laboratory (Platek et al., 2003; Gallup & Gallup Jr, 2007), online (Bartholomew & Cirulli, 2014; Gallup & Wozny, 2023), or the field (*e.g.*, Helt et al., 2010; Massen et al., 2014; Eldakar et al., 2015), although some recent studies have reported a lower percentage (∼15–20%) (Franzen, Mader & Winter, 2018; De Weck et al., 2022; Poole & Henderson, 2023; JJM Massen, TS Roth, REM de Vries, K Dusch, K Malone, A Mentink, ESJ van Dijk, AC Gallup, 2026, unpublished data). Observations of real-world contagious yawning show similar response rates to experimental trials with repeated yawn exposures, particularly when occurring among friends or family (Norscia & Palagi, 2011; Norscia et al., 2020). Why some people are more likely to catch yawns compared to others remains an active area of investigation.

Over the past few decades, there have been considerable efforts to uncover the factors contributing to the individual differences in contagious yawning. Of particular interest has been the examination of clinical populations (Helt et al., 2010; Brown et al., 2017), developmental trends (Bartholomew & Cirulli, 2014; Cordoni, Favilli & Palagi, 2021), and potential gender differences associated with this form of facial mimicry (Gallup & Massen, 2016; Norscia, Demuru & Palagi, 2016). One such line of inquiry has been the association between contagious yawning and measures—or, in most cases, proxies—of empathic processing (*e.g.*, Platek et al., 2003; Senju et al., 2007; Senju et al., 2009; Helt et al., 2010; Helt et al., 2021; Norscia & Palagi, 2011; Usui et al., 2013; Chan & Tseng, 2017; Gallup et al., 2021). In general, the findings thus far have been a mixed bag (Massen & Gallup, 2017). For example, the link between contagious yawning and empathy could be a product of lower-level sensory features such as social attention and/or yawn detection (Palagi et al., 2020; Gallup, 2021; Palagi et al., 2022). Overall, many topics remain unresolved and/or understudied, making this area ripe for further investigation.

One roadblock to systematically evaluating connections between certain psychological measures and the expression of contagious yawning has been the use of different and highly variable stimulus sets. Collectively, this makes direct comparisons both between studies and across conditions difficult, hindering progress within the field. As a result, in recent years there have been efforts to standardize stimulus sets in the field of psychology (Brodeur et al., 2010; Brodeur, Guérard & Bouras, 2014; Ma, Correll & Wittenbrink, 2015). In a review of the methodology used to elicit contagious yawning in the laboratory, Campbell & de Waal (2010) identified three key variables that routinely differ between stimulus sets: (1) the number of yawns displayed, (2) duration of the yawning clips, and (3) the control stimuli. For example, some studies have exposed participants to six yawns with an average duration of 7 s (Senju et al., 2007), while others have displayed 50 yawns averaging less than 5 s in duration (Gallup & Engert, 2019). Even for stimulus sets where the clips are all the same length, the yawns within can be highly variable in duration, appearance, and authenticity (*e.g.*, Platek et al., 2003). Control stimuli used to compare contagion to baseline (spontaneous) yawning rates also vary from one study to the next (*i.e.,* mouth gaping, no mouth movement, laughing, smiling, face scratching) (Senju et al., 2007; Senju et al., 2009; Giganti & Zilli, 2011; Franzen, Mader & Winter, 2018; Poole & Henderson, 2023), with some studies lacking non-yawning control conditions altogether (Bartholomew & Cirulli, 2014; Gallup & Engert, 2019). Collectively, this makes comparisons both between studies and across conditions difficult, hindering progress within the field. In addition, the actors within these stimulus sets often represent a small and homogenous sample that lacks racial/ethnic diversity, and, in some studies, the stimuli are derived from just one or two (Caucasian) people (see Provine, 1989; Nahab et al., 2009; Abe et al., 2015; Norscia et al., 2021). This is problematic since a lack of diversity within psychological stimuli can limit the generalizability of findings beyond the study sample (Chen, Norman & Nam, 2021). Thus, researchers studying contagious yawning would benefit from adopting a single, robust stimulus set.

To address these issues, we developed a free video stimulus set called YawnStim. Compared to existing stimulus sets, YawnStim was designed with greater standardization, ecological validity, and diversity in its representation. Additionally, unlike other stimulus sets, YawnStim provides multiple yawning and non-yawning (control) conditions. Due to the social stigma associated with yawning openly in some Western societies (Schiller, 2002; Brown, Snowden & Gallup, 2022), many people tend to cover their mouths with their hands while yawning. For example, an observational study of a large transit center in Italy showed that 50.8% of male commuters and 67.4% of female commuters actively covered their yawns in public (Schino & Aureli, 1989). Similarly, a more recent laboratory study from the United States reported that college students covered their yawns 44% of time even when alone in a testing room (Gallup & Church, 2015). Considering this backdrop, we created both uncovered and covered yawning conditions. Accompanying these yawning conditions are two control stimuli: mouth closed and mouth gaping conditions. Although smiling and laughing conditions have been previously used as controls for yawning stimuli in some studies (*e.g.*, Giganti & Zilli, 2011; Franzen, Mader & Winter, 2018; Poole & Henderson, 2023), these were not selected due to confounds of emotional salience.

Here, we formally introduce YawnStim and present the results from a series of validating laboratory experiments. To test the effectiveness of the yawning stimuli *versus* the non-yawning controls, each yawning condition was directly compared to both control conditions. In addition, we conducted mini meta-analyses (Goh, Hall & Rosenthal, 2016) to assess the overall effectiveness of the yawning stimuli in eliciting a contagious response and address any inconsistency across the experimental findings. Lastly, for the first time, we compared contagious responses to uncovered yawns and covered yawns.

## Materials & Methods

### Stimulus development

#### Participants

A total of 30 college students at a public university in New York (USA) participated in the development of YawnStim from September 2023 to February 2024. Participants were recruited both through the Introductory Psychology Pool and flyers posted across campus and received either partial course credit or a $20 Amazon gift card in compensation for their time. All participants were 18 years of age or older and enrolled at the university, though their specific age was not obtained. Per the request of the local ethics board, exclusion criteria included citizenship in the European Union due to regulations with human subjects research. Initial recruitment was aimed at acquiring videos from at least two or three male and female participants from each of the following groups based on self-identification: Caucasian, Black, Hispanic/Latino, Middle Eastern, and Asian (note: individuals that did not self-identify within these categories were also allowed to participate in this study). Due to the inability to recruit enough individuals from each category (*i.e.,* Middle Eastern), the final sample (*N* = 24) consisted of three men and three women each self-identifying within one of the following four categories: Caucasian, Black, Hispanic/Latino, and Asian. Videos obtained from all other participants were not retained.

The stimulus development was conducted in accordance with approved human ethics guidelines. All participants provided written consent prior to filming, and video consent was obtained thereafter. In addition, participants signed a video release form permitting the use of their footage in the resulting stimulus package. The Institutional Review Board at SUNY Polytechnic Institute approved the development of this stimulus and its subsequent dissemination to outside researchers (#IRB-Gallup-2022-6).

#### Procedure

The development of the stimulus materials was conducted within a psychology research laboratory. After reviewing and signing the consent form, participants were escorted to a testing area and seated at 60 cm distance from a Canon Vixia HF R42 digital camcorder (3.28 Megapixel Full HD 1,920 × 1,080 resolution) positioned atop a tripod. To standardize the general appearance of individuals within the stimulus, participants wore a black haircut apron over their clothing and sat in front of a white drop cloth background. Prior to recording, each participant was asked to sit comfortably while the researcher modified the height of the tripod to center their face within the video frame. In addition to overhead fluorescent lighting within the testing room, LED photography lights were positioned to each side the participants, producing stable lighting conditions throughout filming: illuminance = 850 LUX (MT-912 light meter).

During each recording session (typically lasting between 15–20 min) participants were instructed to act out each of the following four conditions: (1) mouth closed, (2) mouth gaping, (3) uncovered yawning, and (4) covered yawning. The footage for the mouth closed condition was obtained within a continuous 2-minute span at the beginning of the recording session where participants were asked to look forward at the camera without emotional expression or mouth movement, of which 10 s sequences were extracted. This condition served as a baseline control. Footage for the three action conditions (gaping and yawning) occurred separately and sequentially, with individual recordings that also lasted 10 s in duration. All gaping and yawning actions were instructed to last for 6 s, *i.e.,* average yawn duration in humans (Provine, 1986; Gallup et al., 2016; Massen et al., 2021), with 2 s of footage before and after each display. For the mouth gaping condition, participants were instructed to gape their mouth open while facing the camera. This condition was designed to control for the mouth movement/opening that is characteristic of yawns. Despite this overlap in the motor action pattern of yawning, it was made explicit that these displays were not supposed to be, or look like, yawns. For the two yawning conditions, participants were read aloud the following operational definition to help guide their behavior: “Yawns are an extended gaping of the jaw with inspiration, followed by a brief period of peak muscle contraction and then a passive closure of the jaw with expiration” (adapted from Barbizet, 1958). In the uncovered yawning condition, participants were instructed to act out a yawn as realistically as possible—based on the operational definition and their own experience—while facing the camera. For the covered yawning condition, participants were again asked to perform the same action, but while covering their mouth with one of their hands. To ensure the capture of each targeted behavior in a standardized manner, up to 10 separate recordings were taken for each condition.

#### Stimulus refinement

Once the video acquisition period concluded, the same researcher reviewed each recording and discarded videos that did not meet the basic criteria (*e.g.*, 10 s in duration, where the targeted action is performed to the appropriate duration—6 s—without interruption). The researcher also critically evaluated the yawning trials to ensure the yawns aligned with the operational definition. To discriminate between obviously faked and more realistic yawns, careful attention was paid to subtle changes in head positioning and closure/opening of the eyes during the distinct phases of the yawn. Thus, an effort was made to select the most authentic displays from each participant. During testing, a few participants noted that some trials included actual yawns—probably due to contagion (Provine, 1986)—but in nearly all cases the yawns were performed voluntarily and through coaching by the researcher. Through this guided evaluation, the highest quality clips (that met the inclusion criteria) from each of the four conditions were selected from each of the 24 participants, producing a final stimulus set of 96, 10 s videos. Due to inadvertent ambient noise both within and outside of the laboratory during the recording sessions, and the fact that visual stimuli alone have been shown to be just as effective as combined visual-audio stimuli in eliciting yawn contagion (De Weck et al., 2022), all videos were saved without audio data.

### Stimulus validation

#### Participants

To validate the yawning conditions within YawnStim, *i.e.,* demonstrate that they elicit yawn contagion among observers, a total of 166 college students (120 females and 46 males; mean age: 19.62) were recruited at a private university in Florida (USA) to participate in a study investigating contagious behavior during the 2024–2025 academic year. Data collection began near the end of the Fall 2024 academic term and continued through the entirety of the following Spring 2025 semester with the total sample size determined by recruitment over this period. All participants were at least 18 years old (range: 18–28) and enrolled at the university. Recruitment occurred through the Biology Department and individuals received partial course credit for their participation. Exclusion criteria included taking medications known to alter sleep patterns or mood or a positive history of severe psychiatric or psychological problems. The experiment was conducted in accordance with approved human ethics guidelines, and participants provided written consent prior to the study. The Institutional Review Board at Nova Southeastern University approved this research (#2024-495).

#### Procedure

Experimental testing took place within an office in the biology building, and all sign-ups occurred during the day between 11:00 h and 15:00 h. After reviewing and signing the consent form, participants were seated in front of a 61 cm Dell computer monitor (1,920 × 1,080 resolution) and YawnStim was displayed *via* Windows Media Player 12. Using a within-subjects design, and counterbalancing the presentation order of the stimuli, participants were assigned to one of five experimental comparisons: (1) uncovered yawning *vs.* mouth closed (*N* = 33), (2) uncovered yawning *vs.* mouth gaping (*N* = 33), (3) covered yawning *vs.* mouth closed (*N* = 33), (4) covered yawning *vs.* mouth gaping (*N* = 34), and (5) uncovered yawning *vs.* covered yawning (*N* = 33). Thus, each participant was tested in only one of the five comparisons. Each condition consisted of a randomized compilation of all 24, 10 s video clips, lasting 4-minutes in total duration. The participants were given instructions for viewing the stimulus on the screen and asked to pay close attention to the videos while they were displayed. Since social presence is known to inhibit contagious yawning in laboratory settings (Gallup et al., 2016; Gallup et al., 2019), the researcher left the room prior to testing.

After watching the first stimulus set, participants self-reported on their yawning behavior, which has been replicated to be a valid measure of contagious yawning (Baenninger, 1987; Gallup & Church, 2015; Massen, Church & Gallup, 2015). Participants indicated whether they had yawned at all during testing (dichotomous) and, if so, how many times (yawn frequency). Prior to viewing the second stimulus set, participants also completed a 47-question creativity survey, which was a sham task designed to extend the period between the two stimulus displays and reduce the potential transfer of yawning cues when the yawning stimulus was presented first (Gallup & Wozny, 2025a; Gallup & Wozny, 2025b). Although contagious yawning is a robust phenomenon, the latency can extend for minutes thereafter, and thus most studies have considered yawns to be contagious when they occur within a 3–5-min window following triggering stimuli (Norscia & Palagi, 2011; Kapitány & Nielsen, 2017; Norscia et al., 2020). In this case, the sham survey took roughly 5-min to complete, and additional time was required to complete the self-report yawning measure. After watching the second stimulus set, participants completed the same self-report measure of yawning along with a demographic questionnaire (age, gender).

#### Analysis

A complete distribution of the yawning frequency revealed an extreme outlier in the covered yawning *vs.* mouth gaping comparison, with 24 total yawns (11 and 13, respectively) reported during testing. This exceeded three times the interquartile range, and thus this individual was removed from the dataset prior to the analysis. We used a hurdle approach, whereby separate Generalized Linear Mixed Models (GLMM) were run for the dichotomous measure of yawning (binary logit) and yawn frequency (Poisson), for each of the five experiments. Stimulus condition was included as a fixed effects factor, while participant ID and stimulus presentation order were included as random effects factors. A similar GLMM was also run on the combined data from Experiments 1–4 (*i.e.,* those directly comparing yawning and control conditions) with a Bonferroni correction applied to all *post-hoc* comparisons. In addition, dichotomous and mean differences meta-analyses were conducted on Experiments 1–4 to test the overall effectiveness of the yawning conditions on contagion. All analyses were performed in Jamovi (2.3.21.0), consisting of two-tailed tests with the alpha of 0.05.

## Results

### Experiments 1 & 2: uncovered yawning *vs.* controls

**Figure 1:**
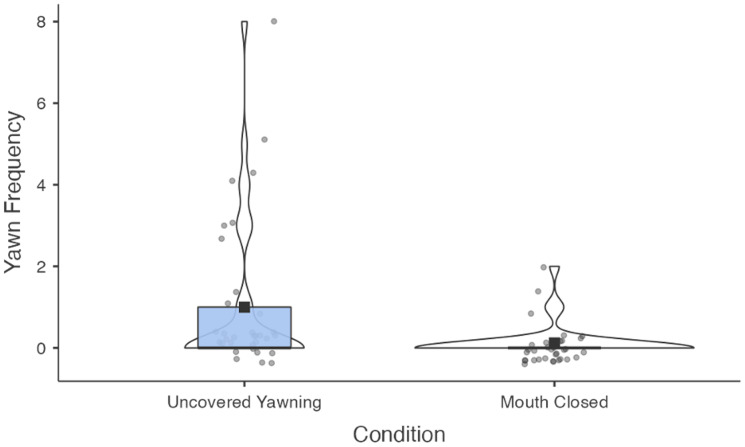
Significantly more yawns were reported during exposure to the uncovered yawning condition compared to the mouth closed condition in Experiment 1. Box plots represent the median, interquartile ranges, and the whiskers extend 1.5 times the interquartile range for the upper and lower boundary (squares are means). Violin plots illustrate the distribution of total yawns (dots are individual participant data).

**Figure 2:**
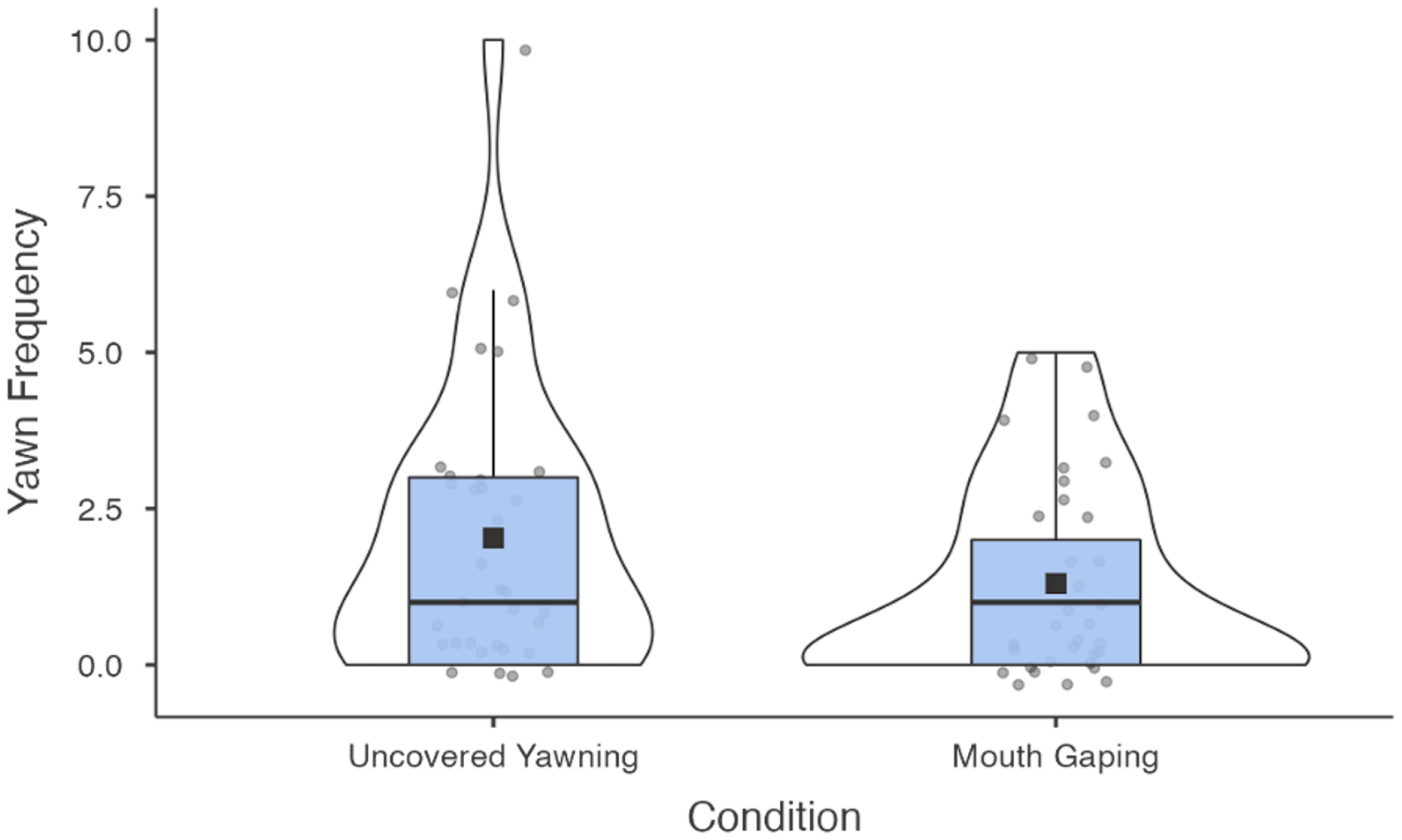
Significantly more yawns were reported during exposure to the uncovered yawning condition compared to the mouth gaping condition in Experiment 2. Box plots represent the median, interquartile ranges, and the whiskers extend 1.5 times the interquartile range for the upper and lower boundary (squares are means). Violin plots illustrate the distribution of total yawns (dots are individual participant data).

The uncovered yawning condition produced an amplified self-reported yawning response in comparison to both control conditions. When compared to the closed mouth control in Experiment 1, participants were significantly more likely to yawn during exposure to the uncovered yawning condition (30.3% *vs.* 9.1%) (Estimate = 1.580 ± SE = 0.743, *X*^2^_(1)_ = 4.50, *p* = 0.034), and reported a significantly greater number of yawns (Estimate = 2.050 ± SE = 0.651, *X*^2^_(1)_ = 9.94, *p* = 0.002; Fig. 1). For Experiment 2, the uncovered yawning condition elicited an enhanced self-reported yawning response relative to the mouth gaping control. Although the dichotomous measure of yawning did not vary between these conditions (Estimate: 0.840 ± SE 0.623, *X*^2^_(1)_ = 1.82, *p* = 0.178), participants reported yawning more often in the uncovered yawning condition (66.7% *vs.* 51.5%). In addition, the total yawn frequency was significantly higher in the uncovered yawning condition (Estimate: 0.459 ± SE 0.195, *X*^2^_(1)_ = 5.55, *p* = 0.018; Fig. 2).

### Experiments 3 & 4: covered yawning *vs.* controls

**Figure 3:**
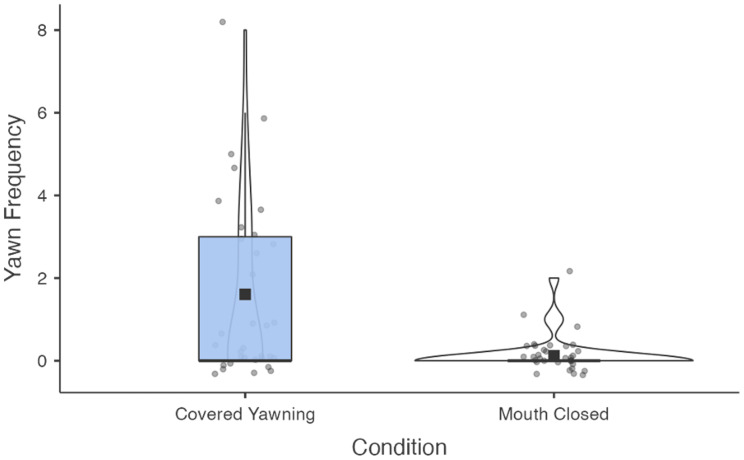
Significantly more yawns were reported during exposure to the covered yawning condition compared to the mouth closed condition in Experiment 3. Box plots represent the median, interquartile ranges, and the whiskers extend 1.5 times the interquartile range for the upper and lower boundary (squares are means). Violin plots illustrate the distribution of total yawns (dots are individual participant data).

**Figure 4:**
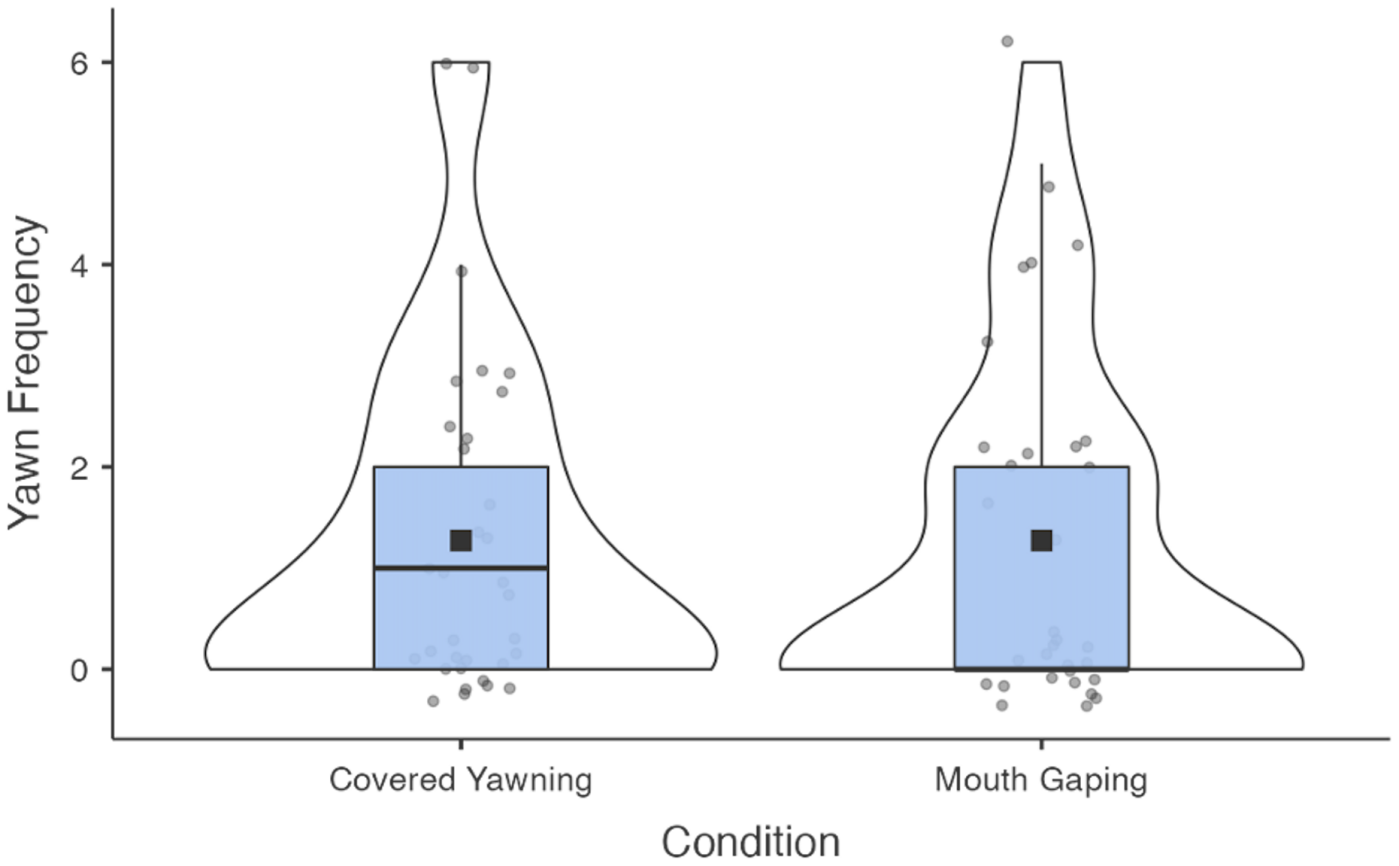
Experiment 4 revealed no significant difference in the total yawns reported during exposure to the covered yawning condition and the mouth gaping condition. Box plots represent the median, interquartile ranges, and the whiskers extend 1.5 times the interquartile range for the upper and lower boundary (squares are means). Violin plots illustrate the distribution of total yawns (dots are individual participant data).

The covered yawning condition activated a stronger yawning response compared to the mouth closed control, but not when compared to the mouth gaping control. Participants in Experiment 3 were significantly more likely to yawn during exposure to the covered yawning condition (48.5% *vs.* 9.1%) (Estimate = 2.600 ± SE = 0.990, *X*^2^_(1)_ = 6.90, *p* = 0.009), and reported a greater number of total yawns (Estimate = 2.560 ± SE = 0.536, *X*^2^_(1)_ = 22.90, *p* < 0.001; Fig. 3). However, results from Experiment 4 showed little to no difference between the covered yawning condition and the mouth gaping control. In fact, no significant effects emerged for either of the two yawning measures: dichotomous (51.5% *vs.* 45.5%) (Estimate = 0.271 ± SE = 0.591, *X*^2^_(1)_ = 0.21, *p* = 0.646); frequency (Estimate = 0.011 ± SE = 0.223, *X*^2^_(1)_ = 0.00, *p* = 0.961; Fig. 4).

### Overall effects of the yawning *vs.* controls

**Figure 5:**
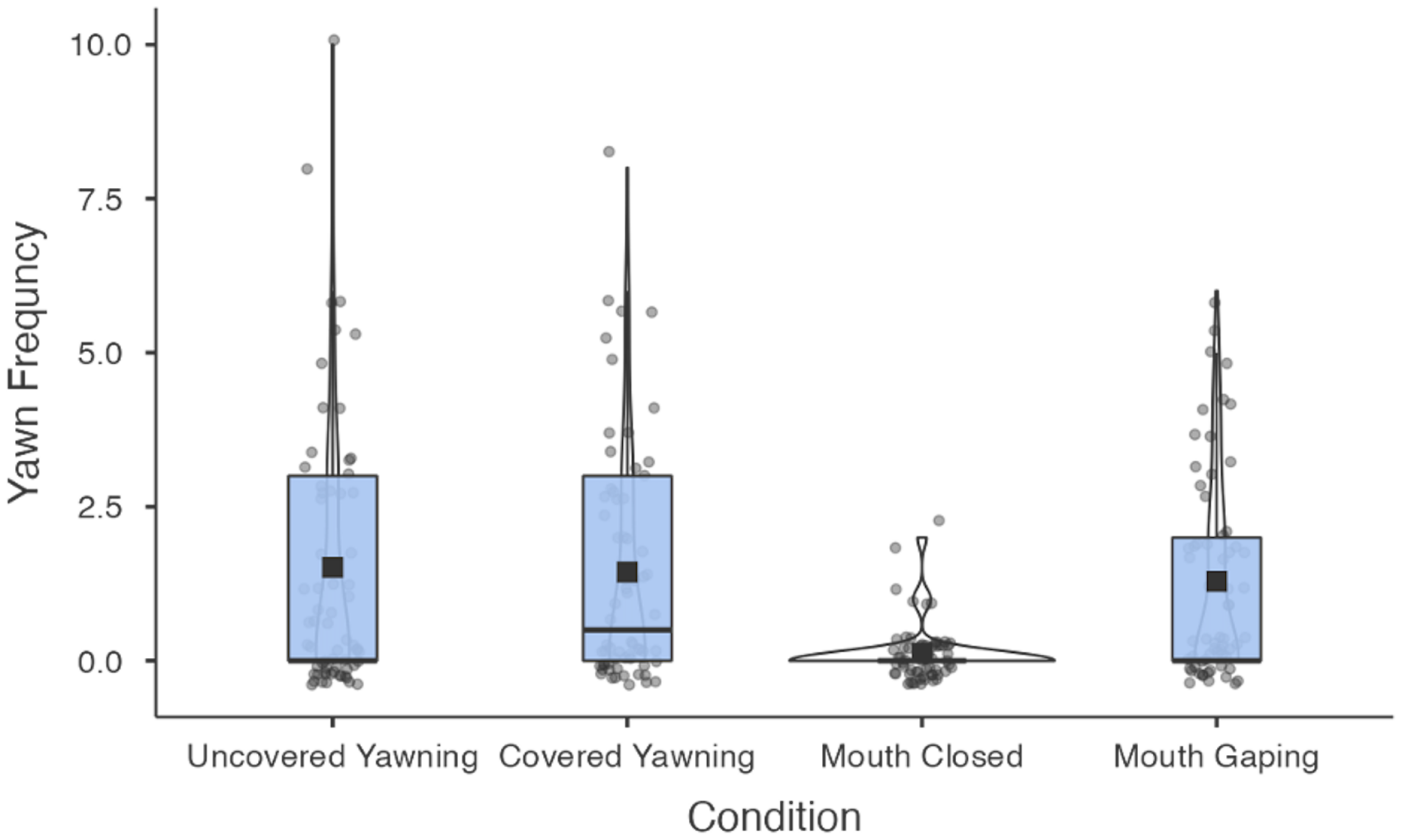
When combining data from Experiments 1–4, there was a significant difference in the total yawns reported across the four conditions. The mouth closed condition elicited fewer yawns compared to all other conditions, which showed a similar yawn frequency. Box plots represent the median, interquartile ranges, and the whiskers extend 1.5 times the interquartile range for the upper and lower boundary (squares are means). Violin plots illustrate the distribution of total yawns (dots are individual participant data).

Descriptive statistics from Experiments 1–4 are presented in Table 1. Overall, there was a significant effect of stimulus condition for both self-reported yawning measures (dichotomous: estimate = 0.733 ± SE = 0.496, *X*^2^_(3)_ = 24.20, *p* < 0.001; frequency: estimate = 0.805 ± SE = 0.310 *X*^2^_(3)_ = 48.20, *p* < 0.001; Fig. 5). *Post-hoc* comparisons revealed that each measure of yawning was significantly lower in the mouth closed control than in all other conditions (*ps* < 0.001). However, neither the dichotomous nor frequency measures of yawning varied significantly between the two yawning conditions and the mouth gaping control (*ps* ≤ 0.851).

**Figure 6:**
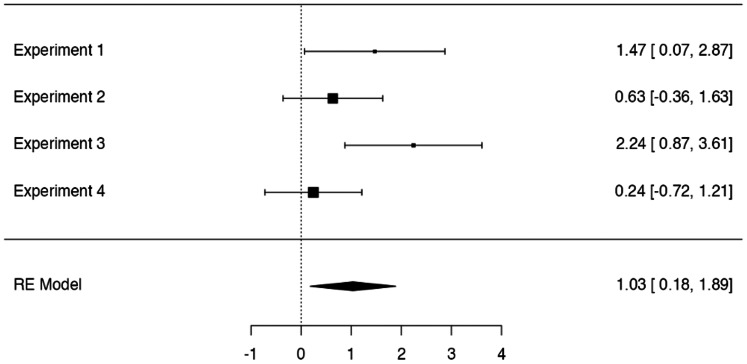
Dichotomous meta-analysis from Experiments 1–4 examining the effect of the yawning conditions on self-reported yawn occurrence. The average outcome was greater than zero (1.03), revealing a significant effect. Effect sizes and 95% CI are presented from each experiment and are represented graphically through the black squares and error bars. Note: Experiment 1 compared the Uncovered Yawning *vs.* Mouth Closed conditions; Experiment 2 compared the Uncovered Yawning *vs.* Mouth Gaping conditions; Experiment 3 compared the Covered Yawning *vs.* Mouth Closed conditions; Experiment 4 compared the Covered Yawning *vs.* Mouth Gaping conditions.

**Figure 7:**
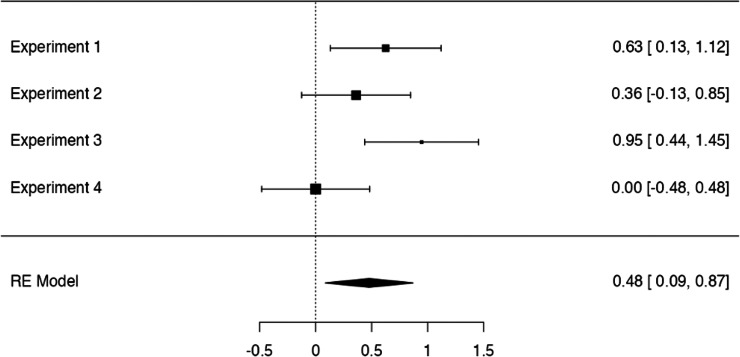
Mean difference meta-analysis from Experiments 1–4 examining the effect of the yawning conditions on self-reported yawn frequency. The average outcome was greater than zero (0.48), revealing a significant effect. Effect sizes and 95% CI are presented from each experiment and are represented graphically through the black squares and error bars. Note: Experiment 1 compared the Uncovered Yawning *vs.* Mouth Closed conditions; Experiment 2 compared the Uncovered Yawning *vs.* Mouth Gaping conditions; Experiment 3 compared the Covered Yawning *vs.* Mouth Closed conditions; Experiment 4 compared the Covered Yawning *vs.* Mouth Gaping conditions.

To more conclusively test the effectiveness of the yawning conditions *versus* the non-yawning controls in eliciting yawns, mini meta-analyses were conducted on Experiments 1–4. For the dichotomous measure, the estimated average log odds ratio based on a random-effects model was 1.034 (95% CI [0.181–1.889]; Fig. 6). This outcome differed significantly from zero (*k* = 4, *Z* = 2.37, *p* = 0.018), demonstrating the effectiveness of the yawning conditions in triggering an initial yawning response. For self-reported yawn frequency, the estimated average standardized mean difference based on the random-effects model was 0.478 (95% CI [0.086–0.870]; Fig. 7). This outcome also differed significantly from zero (*k* = 4, *Z* = 2.39, *p* = 0.017), indicating the yawning conditions were similarly effective in generating more total yawns compared to the controls.

### Experiment 5: uncovered yawning *vs.* covered yawning

**Figure 8:**
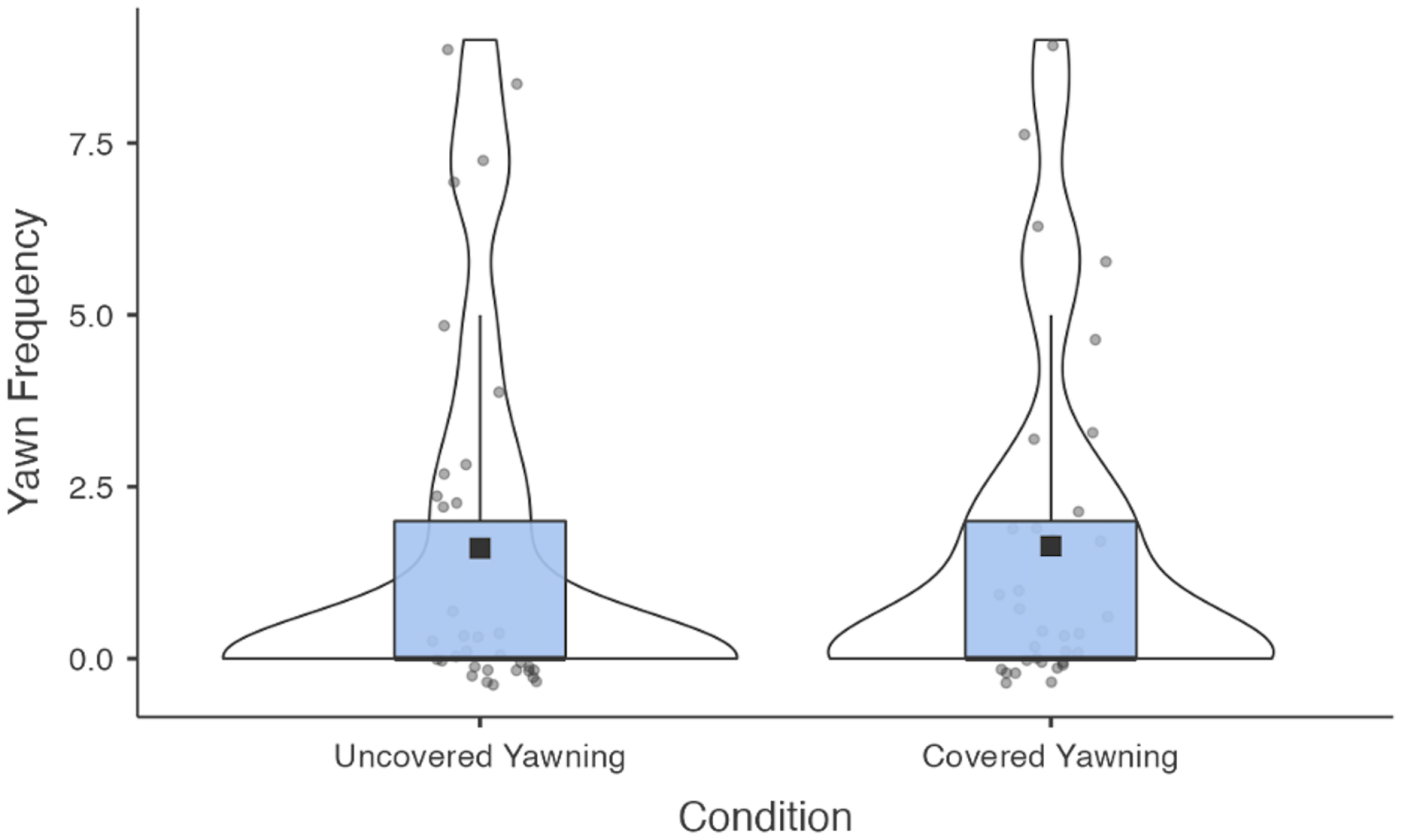
Experiment 5 revealed no significant difference in the total yawns reported during exposure to the uncovered yawning condition and the covered yawning condition. Box plots represent the median, interquartile ranges, and the whiskers extend 1.5 times the interquartile range for the upper and lower boundary (squares are means). Violin plots illustrate the distribution of total yawns (dots are individual participant data).

Lastly, Experiment 5 provided a direct comparison between the uncovered and covered yawning conditions. Results showed a similar yawning response following exposure to each of these displays, as there was neither a significant difference in the proportion of participants yawning (36.4% *vs.* 45.5%) (Estimate: 0.514 ± SE 0.600, *X*^2^_(1)_ = 0.73, *p* = 0.392) nor in the self-reported yawn frequency (Estimate: 0.025 ± SE 0.195, *X*^2^_(1)_ = 0.02, *p* = 0.898; Fig. 8). Thus, both representations of yawning appear equally effective in activating contagion.

## Discussion

The study of contagious yawning in humans has led to important insights into neuropsychological development (Tsurumi, Kanazawa & Yamaguchi, 2019; Cordoni, Favilli & Palagi, 2021; Norscia et al., 2025), clinical conditions (Brown et al., 2017; Helt et al., 2010; Mariscal et al., 2019), social and personality psychology (Bartholomew & Cirulli, 2014; Rundle, Vaughn & Stanford, 2015; Franzen, Mader & Winter, 2018; Gallup et al., 2021; Poole & Henderson, 2023), and neurophysiology and evolutionary biology (Gallup, 2011; Eldakar et al., 2015; Platek, Mohamed & Gallup Jr, 2005; Provine, 2005; Ramirez et al., 2019). Yet, the field has suffered from various methodological issues, particularly the use of different and highly variable stimulus sets across psychological studies (Campbell & de Waal, 2010). This has made it difficult to make meaningful comparisons between studies, and a lack of diversity within existing stimulus sets has hindered the generalizability of the findings from this literature. To specifically address these limitations, YawnStim was developed as a freely available video stimulus set for the scientific community. YawnStim offers advantages over existing stimulus sets by having (1) stable clip duration within and across conditions, (2) uniform filming conditions and clothing/appearance of actors, (3) yawning displays that are minimally variable and ecologically valid in terms of duration, (4) a larger and diverse sample of men and women represented, and (5) multiple yawning and control conditions.

A series of within-subjects laboratory experiments were conducted to test the validity of YawnStim. In addition, this is the first study, to our knowledge, to explicitly compare multiple yawning and non-yawning control displays. As expected, yawning was more common in both the uncovered and covered yawning conditions compared to the mouth closed control condition (Experiments 1 & 3). The uncovered yawning condition also elicited a greater self-reported yawning response compared to the mouth gaping control, though the number of participants that yawned at least once was similar across these conditions (Experiment 2). No differences emerged when comparing the covered yawning condition with the mouth gaping control (Experiment 4), with the latter again producing a heightened yawning response among participants. When combining the data across these four experiments, the uncovered and covered yawning conditions elicited the most yawns, and only a small number of (spontaneous) yawns were observed in the mouth closed control condition. However, no significant differences emerged when comparing either of the yawning conditions to the mouth gaping condition.

**Table 1:** Descriptive statistics from Experiments 1–4 for each of the yawning measures.

	Uncovered Yawning (*N* = 66)	Covered Yawning (*N* = 66)	Mouth Closed (*N* = 66)	Mouth Gaping (*N* = 66)
Yawn Dichotomous	0.485 ± 0.504	0.500 ± 0.504	0.091 ± 0.290	0.485 ± 0.504
Yawn Frequency	1.52 ± 2.18	1.44 ± 1.94	0.12 ± 0.41	1.29 ± 1.65

**Notes.**

Means ± standard deviations are reported.

Mini meta-analyses (Goh, Hall & Rosenthal, 2016) are useful for addressing inconsistency in the results across experiments (Maner, 2014), and thus two such models were conducted including the data from the yawning *versus* non-yawning control comparisons in Experiments 1–4. Based on the output from these models, it can be concluded that the yawning conditions were indeed more effective than the controls in activating a yawning response among participants. Therefore, the overall findings from these laboratory experiments support the validation of YawnStim for studies on contagious yawning.

The relatively high yawning response in the mouth gaping condition was unexpected and is difficult to interpret. One possibility is that despite the subtle, but seemingly important, differences between this condition and the covered and uncovered yawning conditions, the gaping displays simply showed too much of a resemblance to the action pattern of yawning. However, this interpretation challenges previous research indicating that the eye region is an important stimulus for eliciting contagious yawning (Provine, 1989; Senju et al., 2009; Chan & Tseng, 2017), since, unlike the yawning displays, the eyes of participants in the mouth gaping condition remained open throughout the 6 s duration of the action. Alternatively, it is possible that the relatively high rate of yawning during the mouth gaping clips were the result of an uneasiness, stress, or anxiety response, which is known to increase yawning (Provine, 2005; Eldakar et al., 2017). The mouth gaping condition deviated from the other conditions in depicting a relatively unnatural or atypical behavior, and, without solicitation, some participants mentioned to the researchers after testing how they found these clips to be odd or unusual. Further research could be conducted to examine these and other possible explanations for why the mouth gaping condition triggered such a strong effect.

Finally, we offered the first test of how different yawning displays trigger contagion. Results showed that uncovered and covered yawns produce a similar effect (Experiment 5), thus both representations of yawning appear equally effective in activating contagion. In addition, the proportion of participants that reported yawning across all experiments for the uncovered and covered yawning conditions (dichotomous: 30.3%–66.7%) fell squarely within the range of previous studies using a variety of different stimulus sets (*e.g.*, Platek et al., 2003; Gallup & Gallup Jr, 2007; Helt et al., 2010; Usui et al., 2013; Bartholomew & Cirulli, 2014; Massen et al., 2014; De Weck et al., 2022; Eldakar et al., 2017; Franzen, Mader & Winter, 2018; Poole & Henderson, 2023). This is true even if we are to subtract the consistent rate of spontaneous yawning observed in the mouth closed control condition (9.1%). Overall, these findings support the validity of both the uncovered and covered yawning conditions (experimental) as well as the mouth closed condition (control) for future research on contagious yawning.

### Limitations and future research

Limitations to the laboratory experiments should be acknowledged. For one, each condition included all 24, 10 s clips, lasting 4 min in duration. While prior research suggests this is likely an unnecessarily high number of yawning exposures to produce an effect (*e.g.*, Platek et al., 2003; Senju et al., 2007), for the purposes of the validation, we chose to include all men and women from the four racial/ethnic categories represented in each of the stimulus presentations. However, future research should be performed to construct a stimulus response curve to identify the minimal number of yawns sufficient to elicit contagion. Moreover, further research could be undertaken to obtain measures of the perceived authenticity and/or rates of yawn contagion for each uncovered and covered yawning clip. Each 10 s clip within YawnStim has a unique identifier, and data of this nature could be particularly useful in guiding the selection of video compilations used within future studies.

The current research was also limited by not including measures of attention towards the stimulus presentations, which could explain some of the variability in response rates within and across experiments. This is also salient with regards to the mention above regarding the duration of yawning clips. Future research could assess overall visual attention to the yawning and control conditions. The use of eye-tracking could also examine fixations to the eye and/or mouth regions of the actors in each condition (Helt et al., 2021), which could be particularly useful for further assessment of the mouth gaping control. Despite its validity as a measure of contagious yawning (Baenninger, 1987; Massen, Church & Gallup, 2015; Gallup & Church, 2015), the subjective evaluation of yawning could also be considered a limitation. However, a recent follow-up study has also validated YawnStim when capturing video-recordings of yawns, and in doing so revealed a near perfect agreement between self-reported and video confirmed yawns (JJM Massen, TS Roth, REM de Vries, K Dusch, K Malone, A Mentink, ESJ van Dijk, AC Gallup, 2026, unpublished data). Lastly, both the development and validation of YawnStim is limited to a college-aged sample.

## Conclusions

As a standardized and diverse video stimulus, YawnStim has the potential to improve future research on yawn contagion (Provine, 1986), as well as other psychological consequences resulting from the detection of yawns in others. In addition to future investigations into the inter-individual variation in contagious yawning, and its potential connections to empathy (Platek et al., 2003; Massen & Gallup, 2017; Palagi et al., 2020), the unique design of YawnStim lends well to several interesting lines of future research on this phenomenon more generally. The stimuli are particularly well-suited for further study of potential biases in contagious yawning based on sex and in-group/out-group affiliation, whereby stimulus compilations could be customized to participant demographic information (see JJM Massen, TS Roth, REM de Vries, K Dusch, K Malone, A Mentink, ESJ van Dijk, AC Gallup, 2026, unpublished data). Beyond the study of yawn contagion, YawnStim could be used to further examine how merely seeing other people yawn enhances the detection of threatening stimuli (Gallup & Meyers, 2021; Gallup & Wozny, 2025a; Gallup & Wozny, 2025b), and whether this outcome varies as a function of the distinct characteristics of the yawner(s). In addition, due to the stigmatization of yawning in some Western cultures (Brown, Snowden & Gallup, 2022), YawnStim would also be used in the study of affective processes. Therefore, YawnStim has numerous applications.

YawnStim offers significant advantages over existing stimulus sets used in the field. Through a series of laboratory experiments, we demonstrated the robustness and validity of the two yawning conditions in eliciting contagion. If widely adopted, YawnStim would allow for more effective comparisons across studies and laboratories and improve the generalizability of yawning research. YawnStim is free to any interested researchers, and the video files can be obtained electronically by emailing the Principal Investigator and corresponding author (ACG) and providing written agreement to the following terms of use: (1) The video files may be used free of charge for non-commercial, IRB/ethics committee-approved research purposes only; (2) the database materials shall not be re-distributed or published without written consent from the Principal Investigator; (3) no attempts may be made to identify or contact the individuals depicted in the database; (4) the videos will be stored on password protected computers; (5) the videos will not be used for profit; (6) if applicable, compliance with the General Data Protection Regulation of the European Union will be ensured; (7) the videos will not be re-distributed or published without written consent from the Principal Investigator; (8) use of the database materials must be acknowledged in all published work.

## Supplemental Information

10.7717/peerj.21434/supp-1 Supplemental Information 1 Experiment 1 data. raw data; columns labeled

10.7717/peerj.21434/supp-2 Supplemental Information 2 Experiment 2 data. raw data; columns labeled

10.7717/peerj.21434/supp-3 Supplemental Information 3 Experiment 3 data. raw data; columns labeled

10.7717/peerj.21434/supp-4 Supplemental Information 4 Experiment 4 data. raw data; columns labeled

10.7717/peerj.21434/supp-5 Supplemental Information 5 Experiment 5 data. raw data; columns labeled

10.7717/peerj.21434/supp-6 Supplemental Information 6 Meta-analysis data. README

10.7717/peerj.21434/supp-7 Supplemental Information 7 Yawnstim data analysis output.

## Additional Information and Declarations

### Data Availability

The following information was supplied regarding data availability: The data are available at the Harvard Dataverse: Gallup, Andrew, 2025, “YawnStim Laboratory Validation”, https://doi.org/10.7910/DVN/YTEWBC, Harvard Dataverse, V1, UNF:6:QnAM1td2abtvxRDzgUwuxQ== [fileUNF] The raw measurements are available in the Supplemental Files.
