# Supplementary material for: YawnStim: a standardized and diverse video stimulus set
Source: PeerJ. 2026 Jun 23;14:e21434. doi: 10.7717/peerj.21434 (PMC13308540; doi:10.7717/peerj.21434)
Supplement: Supplemental Information 7 [file peerj-14-21434-s007.docx]

Experiment 1: GLMM Output (Yawn Dichotomous)

| Model Info | | | | | |
| --- | --- | --- | --- | --- | --- |
| **Info** | | **Value** | | **Comment** | |
| Model Type |  | Custom |  | Model with custom family |  |
| Call |  | glm |  | Yawn Dichotomous ~ 1 + Condition + (1 \| Order) + (1 \| Participant) |  |
| Link function |  | Logit |  | Log of the odd of y=1 over y=0 |  |
| Distribution |  | Binomial |  | Dichotomous event distribution of y |  |
| LogLikel. |  | -28.462 |  | Unconditional Log-Likelihood |  |
| -2*LogLikel. |  | 56.925 |  | Unconditional absolute deviance |  |
| Deviance |  | 52.422 |  | Conditional relative deviance |  |
| R-squared |  | 0.131 |  | Marginal |  |
| R-squared |  | 0.317 |  | Conditional |  |
| AIC |  | 64.920 |  | Less is better |  |
| BIC |  | 73.683 |  | Less is better |  |
| Residual DF |  | 62.000 |  |  |  |
| Chi-squared/DF |  | 0.835 |  | Overdispersion indicator |  |
| Converged |  | yes |  |  |  |
| Optimizer |  | bobyqa |  |  |  |
| Note. boundary (singular) fit: see ?isSingular | | | | | |
|  | | | | | |

| Fixed Effect Omnibus tests | | | | | | | |
| --- | --- | --- | --- | --- | --- | --- | --- |
|  | | **X²** | | **df** | | **p** | |
| Condition |  | 4.50 |  | 1.00 |  | 0.034 |  |
|  | | | | | | | |

| Fixed Effects Parameter Estimates | | | | | | | | | | | | | | | |
| --- | --- | --- | --- | --- | --- | --- | --- | --- | --- | --- | --- | --- | --- | --- | --- |
|  | | | | | | | | **95% Confidence Interval** | | | |  | | | |
| **Names** | | **Effect** | | **Estimate** | | **SE** | | **Lower** | | **Upper** | | **z** | | **p** | |
| (Intercept) |  | (Intercept) |  | -1.82 |  | 0.794 |  | -3.378 |  | -0.267 |  | -2.30 |  | 0.022 |  |
| Condition1 |  | Uncovered Yawning - Mouth Closed |  | 1.58 |  | 0.743 |  | 0.119 |  | 3.033 |  | 2.12 |  | 0.034 |  |
|  | | | | | | | | | | | | | | | |

| Random Components | | | | | | | | | | | | | |
| --- | --- | --- | --- | --- | --- | --- | --- | --- | --- | --- | --- | --- | --- |
|  | | | | | | | | **Variance 95% C.I.** | | | |  | |
| **Groups** | | **Name** | | **SD** | | **Variance** | | **Lower** | | **Upper** | | **ICC** | |
| Participant |  | (Intercept) |  | 0.000 |  | 0.000 |  | 0.00 |  | 3.89 |  | 0.000 |  |
| Order |  | (Intercept) |  | 0.945 |  | 0.894 |  | NaN |  | NaN |  | 0.214 |  |
| Residuals |  |  |  | 1.000 |  | 1.000 |  | . |  | . |  | . |  |
| Note. Number of Obs: 66 , groups: Participant 33, Order 2 | | | | | | | | | | | | | |
|  | | | | | | | | | | | | | |

Experiment 1: GLMM Output (Yawn Frequency)

| Model Info | | | | | |
| --- | --- | --- | --- | --- | --- |
| **Info** | | **Value** | | **Comment** | |
| Model Type |  | Poisson |  | Model for count data |  |
| Call |  | glm |  | Yawn Frequency ~ 1 + Condition + (1 \| Order) + (1 \| Participant) |  |
| Link function |  | log |  | Coefficients are in the log(y) scale |  |
| Distribution |  | Poisson |  | Model for count data |  |
| LogLikel. |  | -52.454 |  | Unconditional Log-Likelihood |  |
| -2*LogLikel. |  | 104.907 |  | Unconditional absolute deviance |  |
| Deviance |  | 25.545 |  | Conditional relative deviance |  |
| R-squared |  | 0.158 |  | Marginal |  |
| R-squared |  | 0.639 |  | Conditional |  |
| AIC |  | 112.910 |  | Less is better |  |
| BIC |  | 121.666 |  | Less is better |  |
| Residual DF |  | 62.000 |  |  |  |
| Chi-squared/DF |  | 0.350 |  | Overdispersion indicator |  |
| Converged |  | yes |  |  |  |
| Optimizer |  | bobyqa |  |  |  |
|  | | | | | |

| Fixed Effect Omnibus tests | | | | | | | |
| --- | --- | --- | --- | --- | --- | --- | --- |
|  | | **X²** | | **df** | | **p** | |
| Condition |  | 9.94 |  | 1.00 |  | 0.002 |  |
|  | | | | | | | |

| Fixed Effects Parameter Estimates | | | | | | | | | | | | | | | | | | | | | |
| --- | --- | --- | --- | --- | --- | --- | --- | --- | --- | --- | --- | --- | --- | --- | --- | --- | --- | --- | --- | --- | --- |
|  | | | | | | | | **95% Confidence Interval** | | | |  | | **95% Exp(B) Confidence Interval** | | | |  | | | |
| **Names** | | **Effect** | | **Estimate** | | **SE** | | **Lower** | | **Upper** | | **exp(B)** | | **Lower** | | **Upper** | | **z** | | **p** | |
| (Intercept) |  | (Intercept) |  | -2.41 |  | 1.115 |  | -4.593 |  | -0.221 |  | 0.0901 |  | 0.0101 |  | 0.802 |  | -2.16 |  | 0.031 |  |
| Condition1 |  | Uncovered Yawning - Mouth Closed |  | 2.05 |  | 0.651 |  | 0.776 |  | 3.328 |  | 7.7838 |  | 2.1734 |  | 27.877 |  | 3.15 |  | 0.002 |  |
|  | | | | | | | | | | | | | | | | | | | | | |

| Random Components | | | | | | | | | | | | | |
| --- | --- | --- | --- | --- | --- | --- | --- | --- | --- | --- | --- | --- | --- |
|  | | | | | | | | **Variance 95% C.I.** | | | |  | |
| **Groups** | | **Name** | | **SD** | | **Variance** | | **Lower** | | **Upper** | | **ICC** | |
| Participant |  | (Intercept) |  | 1.21 |  | 1.47 |  | 0.3763 |  | 5.31 |  | 0.376 |  |
| Order |  | (Intercept) |  | 1.33 |  | 1.78 |  | 0.0254 |  | 36.59 |  | 0.421 |  |
| Residuals |  |  |  | 1.00 |  | 1.00 |  | . |  | . |  | . |  |
| Note. Number of Obs: 66 , groups: Participant 33, Order 2 | | | | | | | | | | | | | |
|  | | | | | | | | | | | | | |

Experiment 2: GLMM Output (Yawn Dichotomous)

| Model Info | | | | | |
| --- | --- | --- | --- | --- | --- |
| **Info** | | **Value** | | **Comment** | |
| Model Type |  | Custom |  | Model with custom family |  |
| Call |  | glm |  | Yawn Dichotomous ~ 1 + Condition + (1 \| Order) + (1 \| Participant) |  |
| Link function |  | Logit |  | Log of the odd of y=1 over y=0 |  |
| Distribution |  | Binomial |  | Dichotomous event distribution of y |  |
| LogLikel. |  | -43.0929 |  | Unconditional Log-Likelihood |  |
| -2*LogLikel. |  | 86.1857 |  | Unconditional absolute deviance |  |
| Deviance |  | 61.6551 |  | Conditional relative deviance |  |
| R-squared |  | 0.0377 |  | Marginal |  |
| R-squared |  | 0.3085 |  | Conditional |  |
| AIC |  | 94.1900 |  | Less is better |  |
| BIC |  | 102.9443 |  | Less is better |  |
| Residual DF |  | 62.0000 |  |  |  |
| Chi-squared/DF |  | 0.6753 |  | Overdispersion indicator |  |
| Converged |  | yes |  |  |  |
| Optimizer |  | bobyqa |  |  |  |
|  | | | | | |

| Fixed Effect Omnibus tests | | | | | | | |
| --- | --- | --- | --- | --- | --- | --- | --- |
|  | | **X²** | | **df** | | **p** | |
| Condition |  | 1.82 |  | 1.00 |  | 0.178 |  |
|  | | | | | | | |

| Fixed Effects Parameter Estimates | | | | | | | | | | | | | | | | | | | | | |
| --- | --- | --- | --- | --- | --- | --- | --- | --- | --- | --- | --- | --- | --- | --- | --- | --- | --- | --- | --- | --- | --- |
|  | | | | | | | | **95% Confidence Interval** | | | |  | | **95% Exp(B) Confidence Interval** | | | |  | | | |
| **Names** | | **Effect** | | **Estimate** | | **SE** | | **Lower** | | **Upper** | | **exp(B)** | | **Lower** | | **Upper** | | **z** | | **p** | |
| (Intercept) |  | (Intercept) |  | 0.480 |  | 0.469 |  | -0.440 |  | 1.40 |  | 1.62 |  | 0.644 |  | 4.06 |  | 1.02 |  | 0.306 |  |
| Condition1 |  | Uncovered Yawning - Mouth Gaping |  | 0.840 |  | 0.623 |  | -0.382 |  | 2.06 |  | 2.32 |  | 0.683 |  | 7.86 |  | 1.35 |  | 0.178 |  |
|  | | | | | | | | | | | | | | | | | | | | | |

| Random Components | | | | | | | | | |
| --- | --- | --- | --- | --- | --- | --- | --- | --- | --- |
| **Groups** | | **Name** | | **SD** | | **Variance** | | **ICC** | |
| Participant |  | (Intercept) |  | 1.052 |  | 1.107 |  | 0.2517 |  |
| Order |  | (Intercept) |  | 0.426 |  | 0.182 |  | 0.0523 |  |
| Residuals |  |  |  | 1.000 |  | 1.000 |  | . |  |
| Note. Number of Obs: 66 , groups: Participant 33, Order 2 | | | | | | | | | |
|  | | | | | | | | | |

Experiment 2: GLMM Output (Yawn Frequency)

| Model Info | | | | | |
| --- | --- | --- | --- | --- | --- |
| **Info** | | **Value** | | **Comment** | |
| Model Type |  | Poisson |  | Model for count data |  |
| Call |  | glm |  | Yawn Frequency ~ 1 + Condition + (1 \| Order) + (1 \| Participant) |  |
| Link function |  | log |  | Coefficients are in the log(y) scale |  |
| Distribution |  | Poisson |  | Model for count data |  |
| LogLikel. |  | -116.9517 |  | Unconditional Log-Likelihood |  |
| -2*LogLikel. |  | 233.9035 |  | Unconditional absolute deviance |  |
| Deviance |  | 71.2934 |  | Conditional relative deviance |  |
| R-squared |  | 0.0406 |  | Marginal |  |
| R-squared |  | 0.5473 |  | Conditional |  |
| AIC |  | 241.9000 |  | Less is better |  |
| BIC |  | 250.6621 |  | Less is better |  |
| Residual DF |  | 62.0000 |  |  |  |
| Chi-squared/DF |  | 0.9055 |  | Overdispersion indicator |  |
| Converged |  | yes |  |  |  |
| Optimizer |  | bobyqa |  |  |  |
|  | | | | | |

| Fixed Effect Omnibus tests | | | | | | | |
| --- | --- | --- | --- | --- | --- | --- | --- |
|  | | **X²** | | **df** | | **p** | |
| Condition |  | 5.55 |  | 1.00 |  | 0.018 |  |
|  | | | | | | | |

| Fixed Effects Parameter Estimates | | | | | | | | | | | | | | | | | |
| --- | --- | --- | --- | --- | --- | --- | --- | --- | --- | --- | --- | --- | --- | --- | --- | --- | --- |
|  | | | | | | | | | | **95% Exp(B) Confidence Interval** | | | |  | | | |
| **Names** | | **Effect** | | **Estimate** | | **SE** | | **exp(B)** | | **Lower** | | **Upper** | | **z** | | **p** | |
| (Intercept) |  | (Intercept) |  | 0.183 |  | 0.307 |  | 1.20 |  | 0.657 |  | 2.19 |  | 0.596 |  | 0.551 |  |
| Condition1 |  | Uncovered Yawning - Mouth Gaping |  | 0.459 |  | 0.195 |  | 1.58 |  | 1.080 |  | 2.32 |  | 2.357 |  | 0.018 |  |
|  | | | | | | | | | | | | | | | | | |

| Random Components | | | | | | | | | | | | | |
| --- | --- | --- | --- | --- | --- | --- | --- | --- | --- | --- | --- | --- | --- |
|  | | | | | | | | **Variance 95% C.I.** | | | |  | |
| **Groups** | | **Name** | | **SD** | | **Variance** | | **Lower** | | **Upper** | | **ICC** | |
| Participant |  | (Intercept) |  | 0.738 |  | 0.544 |  | 0.1892 |  | 1.41 |  | 0.478 |  |
| Order |  | (Intercept) |  | 0.349 |  | 0.122 |  | 0.0113 |  | 2.49 |  | 0.170 |  |
| Residuals |  |  |  | 1.000 |  | 1.000 |  | . |  | . |  | . |  |
| Note. Number of Obs: 66 , groups: Participant 33, Order 2 | | | | | | | | | | | | | |
|  | | | | | | | | | | | | | |

Experiment 3: GLMM Output (Yawn Dichotomous)

| Model Info | | | | | |
| --- | --- | --- | --- | --- | --- |
| **Info** | | **Value** | | **Comment** | |
| Model Type |  | Custom |  | Model with custom family |  |
| Call |  | glm |  | Yawn Dichotomous ~ 1 + Condition + (1 \| Order) + (1 \| Participant) |  |
| Link function |  | Logit |  | Log of the odd of y=1 over y=0 |  |
| Distribution |  | Binomial |  | Dichotomous event distribution of y |  |
| LogLikel. |  | -32.415 |  | Unconditional Log-Likelihood |  |
| -2*LogLikel. |  | 64.830 |  | Unconditional absolute deviance |  |
| Deviance |  | 49.364 |  | Conditional relative deviance |  |
| R-squared |  | 0.283 |  | Marginal |  |
| R-squared |  | 0.458 |  | Conditional |  |
| AIC |  | 72.830 |  | Less is better |  |
| BIC |  | 81.589 |  | Less is better |  |
| Residual DF |  | 62.000 |  |  |  |
| Chi-squared/DF |  | 0.704 |  | Overdispersion indicator |  |
| Converged |  | yes |  |  |  |
| Optimizer |  | bobyqa |  |  |  |
|  | | | | | |

| Fixed Effect Omnibus tests | | | | | | | |
| --- | --- | --- | --- | --- | --- | --- | --- |
|  | | **X²** | | **df** | | **p** | |
| Condition |  | 6.90 |  | 1.00 |  | 0.009 |  |
|  | | | | | | | |

| Fixed Effects Parameter Estimates | | | | | | | | | | | | | | | |
| --- | --- | --- | --- | --- | --- | --- | --- | --- | --- | --- | --- | --- | --- | --- | --- |
|  | | | | | | | | **95% Confidence Interval** | | | |  | | | |
| **Names** | | **Effect** | | **Estimate** | | **SE** | | **Lower** | | **Upper** | | **z** | | **p** | |
| (Intercept) |  | (Intercept) |  | -1.38 |  | 0.642 |  | -2.64 |  | -0.121 |  | -2.15 |  | 0.032 |  |
| Condition1 |  | Mouth Closed - Covered Yawning |  | -2.60 |  | 0.990 |  | -4.54 |  | -0.659 |  | -2.63 |  | 0.009 |  |
|  | | | | | | | | | | | | | | | |

| Random Components | | | | | | | | | | | | | |
| --- | --- | --- | --- | --- | --- | --- | --- | --- | --- | --- | --- | --- | --- |
|  | | | | | | | | **Variance 95% C.I.** | | | |  | |
| **Groups** | | **Name** | | **SD** | | **Variance** | | **Lower** | | **Upper** | | **ICC** | |
| Participant |  | (Intercept) |  | 0.890 |  | 0.793 |  | 0.00 |  | 26.0 |  | 0.1942 |  |
| Order |  | (Intercept) |  | 0.522 |  | 0.273 |  | 0.00 |  | 10.6 |  | 0.0766 |  |
| Residuals |  |  |  | 1.000 |  | 1.000 |  | . |  | . |  | . |  |
| Note. Number of Obs: 66 , groups: Participant 33, Order 2 | | | | | | | | | | | | | |
|  | | | | | | | | | | | | | |

Experiment 3: GLMM Output (Yawn Frequency)

| Model Info | | | | | |
| --- | --- | --- | --- | --- | --- |
| **Info** | | **Value** | | **Comment** | |
| Model Type |  | Poisson |  | Model for count data |  |
| Call |  | glm |  | Yawn Frequency ~ 1 + Condition + (1 \| Order) + (1 \| Participant) |  |
| Link function |  | log |  | Coefficients are in the log(y) scale |  |
| Distribution |  | Poisson |  | Model for count data |  |
| LogLikel. |  | -69.113 |  | Unconditional Log-Likelihood |  |
| -2*LogLikel. |  | 138.226 |  | Unconditional absolute deviance |  |
| Deviance |  | 29.941 |  | Conditional relative deviance |  |
| R-squared |  | 0.357 |  | Marginal |  |
| R-squared |  | 0.722 |  | Conditional |  |
| AIC |  | 146.230 |  | Less is better |  |
| BIC |  | 154.985 |  | Less is better |  |
| Residual DF |  | 62.000 |  |  |  |
| Chi-squared/DF |  | 0.446 |  | Overdispersion indicator |  |
| Converged |  | yes |  |  |  |
| Optimizer |  | bobyqa |  |  |  |
|  | | | | | |

| Fixed Effect Omnibus tests | | | | | | | |
| --- | --- | --- | --- | --- | --- | --- | --- |
|  | | **X²** | | **df** | | **p** | |
| Condition |  | 22.9 |  | 1.00 |  | < .001 |  |
|  | | | | | | | |

| Fixed Effects Parameter Estimates | | | | | | | | | | | | | | | | | | | | | |
| --- | --- | --- | --- | --- | --- | --- | --- | --- | --- | --- | --- | --- | --- | --- | --- | --- | --- | --- | --- | --- | --- |
|  | | | | | | | | **95% Confidence Interval** | | | |  | | **95% Exp(B) Confidence Interval** | | | |  | | | |
| **Names** | | **Effect** | | **Estimate** | | **SE** | | **Lower** | | **Upper** | | **exp(B)** | | **Lower** | | **Upper** | | **z** | | **p** | |
| (Intercept) |  | (Intercept) |  | -1.56 |  | 0.530 |  | -2.60 |  | -0.517 |  | 0.2108 |  | 0.0745 |  | 0.596 |  | -2.93 |  | 0.003 |  |
| Condition1 |  | Mouth Closed - Covered Yawning |  | -2.56 |  | 0.536 |  | -3.62 |  | -1.513 |  | 0.0770 |  | 0.0269 |  | 0.220 |  | -4.78 |  | < .001 |  |
|  | | | | | | | | | | | | | | | | | | | | | |

| Random Components | | | | | | | | | | | | | |
| --- | --- | --- | --- | --- | --- | --- | --- | --- | --- | --- | --- | --- | --- |
|  | | | | | | | | **Variance 95% C.I.** | | | |  | |
| **Groups** | | **Name** | | **SD** | | **Variance** | | **Lower** | | **Upper** | | **ICC** | |
| Participant |  | (Intercept) |  | 1.234 |  | 1.523 |  | 0.506 |  | 4.45 |  | 0.539 |  |
| Order |  | (Intercept) |  | 0.430 |  | 0.185 |  | 0.000 |  | 4.69 |  | 0.125 |  |
| Residuals |  |  |  | 1.000 |  | 1.000 |  | . |  | . |  | . |  |
| Note. Number of Obs: 66 , groups: Participant 33, Order 2 | | | | | | | | | | | | | |
|  | | | | | | | | | | | | | |

Experiment 4: GLMM Output (Yawn Dichotomous)

| Model Info | | | | | |
| --- | --- | --- | --- | --- | --- |
| **Info** | | **Value** | | **Comment** | |
| Model Type |  | Custom |  | Model with custom family |  |
| Call |  | glm |  | Yawn Dichotomous ~ 1 + Condition + (1 \| Order) + (1 \| Participant) |  |
| Link function |  | Logit |  | Log of the odd of y=1 over y=0 |  |
| Distribution |  | Binomial |  | Dichotomous event distribution of y |  |
| LogLikel. |  | -44.23241 |  | Unconditional Log-Likelihood |  |
| -2*LogLikel. |  | 88.46482 |  | Unconditional absolute deviance |  |
| Deviance |  | 55.71692 |  | Conditional relative deviance |  |
| R-squared |  | 0.00347 |  | Marginal |  |
| R-squared |  | 0.38766 |  | Conditional |  |
| AIC |  | 96.46000 |  | Less is better |  |
| BIC |  | 105.22344 |  | Less is better |  |
| Residual DF |  | 62.00000 |  |  |  |
| Chi-squared/DF |  | 0.58581 |  | Overdispersion indicator |  |
| Converged |  | yes |  |  |  |
| Optimizer |  | bobyqa |  |  |  |
|  | | | | | |

| Fixed Effect Omnibus tests | | | | | | | |
| --- | --- | --- | --- | --- | --- | --- | --- |
|  | | **X²** | | **df** | | **p** | |
| Condition |  | 0.211 |  | 1.00 |  | 0.646 |  |
|  | | | | | | | |

| Fixed Effects Parameter Estimates | | | | | | | | | | | | | | | | | | | | | |
| --- | --- | --- | --- | --- | --- | --- | --- | --- | --- | --- | --- | --- | --- | --- | --- | --- | --- | --- | --- | --- | --- |
|  | | | | | | | | **95% Confidence Interval** | | | |  | | **95% Exp(B) Confidence Interval** | | | |  | | | |
| **Names** | | **Effect** | | **Estimate** | | **SE** | | **Lower** | | **Upper** | | **exp(B)** | | **Lower** | | **Upper** | | **z** | | **p** | |
| (Intercept) |  | (Intercept) |  | -0.0868 |  | 0.535 |  | -1.14 |  | 0.962 |  | 0.917 |  | 0.321 |  | 2.62 |  | -0.162 |  | 0.871 |  |
| Condition1 |  | Mouth Gaping - Covered Yawning |  | -0.2712 |  | 0.591 |  | -1.43 |  | 0.886 |  | 0.762 |  | 0.240 |  | 2.43 |  | -0.459 |  | 0.646 |  |
|  | | | | | | | | | | | | | | | | | | | | | |

| Random Components | | | | | | | | | | | | | |
| --- | --- | --- | --- | --- | --- | --- | --- | --- | --- | --- | --- | --- | --- |
|  | | | | | | | | **Variance 95% C.I.** | | | |  | |
| **Groups** | | **Name** | | **SD** | | **Variance** | | **Lower** | | **Upper** | | **ICC** | |
| Participant |  | (Intercept) |  | 1.331 |  | 1.771 |  | 0.00 |  | 11.35 |  | 0.3500 |  |
| Order |  | (Intercept) |  | 0.541 |  | 0.293 |  | 0.00 |  | 9.30 |  | 0.0818 |  |
| Residuals |  |  |  | 1.000 |  | 1.000 |  | . |  | . |  | . |  |
| Note. Number of Obs: 66 , groups: Participant 33, Order 2 | | | | | | | | | | | | | |
|  | | | | | | | | | | | | | |

Experiment 4: GLMM Output (Yawn Frequency)

| Model Info | | | | | |
| --- | --- | --- | --- | --- | --- |
| **Info** | | **Value** | | **Comment** | |
| Model Type |  | Poisson |  | Model for count data |  |
| Call |  | glm |  | Yawn Frequency ~ 1 + Condition + (1 \| Order) + (1 \| Participant) |  |
| Link function |  | log |  | Coefficients are in the log(y) scale |  |
| Distribution |  | Poisson |  | Model for count data |  |
| LogLikel. |  | -101.610 |  | Unconditional Log-Likelihood |  |
| -2*LogLikel. |  | 203.220 |  | Unconditional absolute deviance |  |
| Deviance |  | 55.723 |  | Conditional relative deviance |  |
| R-squared |  | 1.56e-5 |  | Marginal |  |
| R-squared |  | 0.578 |  | Conditional |  |
| AIC |  | 211.220 |  | Less is better |  |
| BIC |  | 219.979 |  | Less is better |  |
| Residual DF |  | 62.000 |  |  |  |
| Chi-squared/DF |  | 0.663 |  | Overdispersion indicator |  |
| Converged |  | yes |  |  |  |
| Optimizer |  | bobyqa |  |  |  |
|  | | | | | |

| Fixed Effect Omnibus tests | | | | | | | |
| --- | --- | --- | --- | --- | --- | --- | --- |
|  | | **X²** | | **df** | | **p** | |
| Condition |  | 0.00243 |  | 1.00 |  | 0.961 |  |
|  | | | | | | | |

| Fixed Effects Parameter Estimates | | | | | | | | | | | | | | | | | |
| --- | --- | --- | --- | --- | --- | --- | --- | --- | --- | --- | --- | --- | --- | --- | --- | --- | --- |
|  | | | | | | | | | | **95% Exp(B) Confidence Interval** | | | |  | | | |
| **Names** | | **Effect** | | **Estimate** | | **SE** | | **exp(B)** | | **Lower** | | **Upper** | | **z** | | **p** | |
| (Intercept) |  | (Intercept) |  | -0.2591 |  | 0.325 |  | 0.772 |  | 0.408 |  | 1.46 |  | -0.7959 |  | 0.426 |  |
| Condition1 |  | Mouth Gaping - Covered Yawning |  | 0.0110 |  | 0.223 |  | 1.011 |  | 0.653 |  | 1.57 |  | 0.0493 |  | 0.961 |  |
|  | | | | | | | | | | | | | | | | | |

| Random Components | | | | | | | | | | | | | |
| --- | --- | --- | --- | --- | --- | --- | --- | --- | --- | --- | --- | --- | --- |
|  | | | | | | | | **Variance 95% C.I.** | | | |  | |
| **Groups** | | **Name** | | **SD** | | **Variance** | | **Lower** | | **Upper** | | **ICC** | |
| Participant |  | (Intercept) |  | 1.031 |  | 1.0631 |  | 0.408 |  | 2.71 |  | 0.5612 |  |
| Order |  | (Intercept) |  | 0.271 |  | 0.0737 |  | 0.000 |  | 1.71 |  | 0.0814 |  |
| Residuals |  |  |  | 1.000 |  | 1.0000 |  | . |  | . |  | . |  |
| Note. Number of Obs: 66 , groups: Participant 33, Order 2 | | | | | | | | | | | | | |
|  | | | | | | | | | | | | | |

Combined Results (Experiments 1-4): GLMM Output (Yawn Dichotomous)

| Model Info | | | | | |
| --- | --- | --- | --- | --- | --- |
| **Info** | | **Value** | | **Comment** | |
| Model Type |  | Custom |  | Model with custom family |  |
| Call |  | glm |  | Yawn Dichotomous ~ 1 + Condition + (1 \| Order) + (1 \| Participant) |  |
| Link function |  | Logit |  | Log of the odd of y=1 over y=0 |  |
| Distribution |  | Binomial |  | Dichotomous event distribution of y |  |
| LogLikel. |  | -150.389 |  | Unconditional Log-Likelihood |  |
| -2*LogLikel. |  | 300.778 |  | Unconditional absolute deviance |  |
| Deviance |  | 225.626 |  | Conditional relative deviance |  |
| R-squared |  | 0.217 |  | Marginal |  |
| R-squared |  | 0.444 |  | Conditional |  |
| AIC |  | 312.780 |  | Less is better |  |
| BIC |  | 334.234 |  | Less is better |  |
| Residual DF |  | 258.000 |  |  |  |
| Chi-squared/DF |  | 0.665 |  | Overdispersion indicator |  |
| Converged |  | yes |  |  |  |
| Optimizer |  | bobyqa |  |  |  |
|  | | | | | |

| Fixed Effect Omnibus tests | | | | | | | |
| --- | --- | --- | --- | --- | --- | --- | --- |
|  | | **X²** | | **df** | | **p** | |
| Condition |  | 24.2 |  | 3.00 |  | < .001 |  |
|  | | | | | | | |

| Fixed Effects Parameter Estimates | | | | | | | | | | | | | | | | | | | | | |
| --- | --- | --- | --- | --- | --- | --- | --- | --- | --- | --- | --- | --- | --- | --- | --- | --- | --- | --- | --- | --- | --- |
|  | | | | | | | | **95% Confidence Interval** | | | |  | | **95% Exp(B) Confidence Interval** | | | |  | | | |
| **Names** | | **Effect** | | **Estimate** | | **SE** | | **Lower** | | **Upper** | | **exp(B)** | | **Lower** | | **Upper** | | **z** | | **p** | |
| (Intercept) |  | (Intercept) |  | -0.733 |  | 0.496 |  | -1.71 |  | 0.239 |  | 0.480 |  | 0.182 |  | 1.27 |  | -1.48 |  | 0.139 |  |
| Condition1 |  | Uncovered Yawning - Mouth Closed |  | 2.611 |  | 0.585 |  | 1.46 |  | 3.758 |  | 13.613 |  | 4.323 |  | 42.86 |  | 4.46 |  | < .001 |  |
| Condition2 |  | Mouth Gaping - Mouth Closed |  | 2.554 |  | 0.580 |  | 1.42 |  | 3.690 |  | 12.855 |  | 4.125 |  | 40.06 |  | 4.40 |  | < .001 |  |
| Condition3 |  | Covered Yawning - Mouth Closed |  | 2.664 |  | 0.588 |  | 1.51 |  | 3.816 |  | 14.348 |  | 4.531 |  | 45.44 |  | 4.53 |  | < .001 |  |
|  | | | | | | | | | | | | | | | | | | | | | |

| Random Components | | | | | | | | | | | | | |
| --- | --- | --- | --- | --- | --- | --- | --- | --- | --- | --- | --- | --- | --- |
|  | | | | | | | | **Variance 95% C.I.** | | | |  | |
| **Groups** | | **Name** | | **SD** | | **Variance** | | **Lower** | | **Upper** | | **ICC** | |
| Participant |  | (Intercept) |  | 0.967 |  | 0.936 |  | 0.0000 |  | 3.33 |  | 0.221 |  |
| Order |  | (Intercept) |  | 0.642 |  | 0.412 |  | -0.0122 |  | 8.22 |  | 0.111 |  |
| Residuals |  |  |  | 1.000 |  | 1.000 |  | . |  | . |  | . |  |
| Note. Number of Obs: 264 , groups: Participant 132, Order 2 | | | | | | | | | | | | | |
|  | | | | | | | | | | | | | |

Combined Results (Experiments 1-4): GLMM Output (Yawn Frequency)

| Model Info | | | | | |
| --- | --- | --- | --- | --- | --- |
| **Info** | | **Value** | | **Comment** | |
| Model Type |  | Poisson |  | Model for count data |  |
| Call |  | glm |  | Yawn Frequency ~ 1 + Condition + (1 \| Order) + (1 \| Participant) |  |
| Link function |  | log |  | Coefficients are in the log(y) scale |  |
| Distribution |  | Poisson |  | Model for count data |  |
| LogLikel. |  | -345.856 |  | Unconditional Log-Likelihood |  |
| -2*LogLikel. |  | 691.711 |  | Unconditional absolute deviance |  |
| Deviance |  | 198.967 |  | Conditional relative deviance |  |
| R-squared |  | 0.314 |  | Marginal |  |
| R-squared |  | 0.679 |  | Conditional |  |
| AIC |  | 703.710 |  | Less is better |  |
| BIC |  | 725.167 |  | Less is better |  |
| Residual DF |  | 258.000 |  |  |  |
| Chi-squared/DF |  | 0.654 |  | Overdispersion indicator |  |
| Converged |  | yes |  |  |  |
| Optimizer |  | bobyqa |  |  |  |
|  | | | | | |

| Fixed Effect Omnibus tests | | | | | | | |
| --- | --- | --- | --- | --- | --- | --- | --- |
|  | | **X²** | | **df** | | **p** | |
| Condition |  | 48.2 |  | 3.00 |  | < .001 |  |
|  | | | | | | | |

| Fixed Effects Parameter Estimates | | | | | | | | | | | | | | | | | | | | | |
| --- | --- | --- | --- | --- | --- | --- | --- | --- | --- | --- | --- | --- | --- | --- | --- | --- | --- | --- | --- | --- | --- |
|  | | | | | | | | **95% Confidence Interval** | | | |  | | **95% Exp(B) Confidence Interval** | | | |  | | | |
| **Names** | | **Effect** | | **Estimate** | | **SE** | | **Lower** | | **Upper** | | **exp(B)** | | **Lower** | | **Upper** | | **z** | | **p** | |
| (Intercept) |  | (Intercept) |  | -0.805 |  | 0.310 |  | -1.41 |  | -0.198 |  | 0.447 |  | 0.244 |  | 0.820 |  | -2.60 |  | 0.009 |  |
| Condition1 |  | Uncovered Yawning - Mouth Closed |  | 2.431 |  | 0.364 |  | 1.72 |  | 3.144 |  | 11.375 |  | 5.579 |  | 23.193 |  | 6.69 |  | < .001 |  |
| Condition2 |  | Mouth Gaping - Mouth Closed |  | 2.181 |  | 0.367 |  | 1.46 |  | 2.901 |  | 8.858 |  | 4.311 |  | 18.201 |  | 5.94 |  | < .001 |  |
| Condition3 |  | Covered Yawning - Mouth Closed |  | 2.291 |  | 0.356 |  | 1.59 |  | 2.988 |  | 9.887 |  | 4.925 |  | 19.849 |  | 6.44 |  | < .001 |  |
|  | | | | | | | | | | | | | | | | | | | | | |

| Random Components | | | | | | | | | |
| --- | --- | --- | --- | --- | --- | --- | --- | --- | --- |
| **Groups** | | **Name** | | **SD** | | **Variance** | | **ICC** | |
| Participant |  | (Intercept) |  | 1.011 |  | 1.022 |  | 0.498 |  |
| Order |  | (Intercept) |  | 0.384 |  | 0.147 |  | 0.125 |  |
| Residuals |  |  |  | 1.000 |  | 1.000 |  | . |  |
| Note. Number of Obs: 264 , groups: Participant 132, Order 2 | | | | | | | | | |
|  | | | | | | | | | |

Mini Meta-Analysis (Yawn Dichotomous)

| Random-Effects Model (k = 4) | | | | | | | | | | | | | |
| --- | --- | --- | --- | --- | --- | --- | --- | --- | --- | --- | --- | --- | --- |
|  | | **Estimate** | | **se** | | **Z** | | **p** | | **CI Lower Bound** | | **CI Upper Bound** | |
| Intercept |  | 1.03 |  | 0.436 |  | 2.37 |  | 0.018 |  | 0.180 |  | 1.889 |  |
|  |  | . |  | . |  | . |  | . |  | . |  | . |  |
| Note. Tau² Estimator: Restricted Maximum-Likelihood | | | | | | | | | | | | | |
|  | | | | | | | | | | | | | |

Mini Meta-Analysis (Yawn Frequency)

| Random-Effects Model (k = 4) | | | | | | | | | | | | | |
| --- | --- | --- | --- | --- | --- | --- | --- | --- | --- | --- | --- | --- | --- |
|  | | **Estimate** | | **se** | | **Z** | | **p** | | **CI Lower Bound** | | **CI Upper Bound** | |
| Intercept |  | 0.478 |  | 0.200 |  | 2.39 |  | 0.017 |  | 0.086 |  | 0.870 |  |
|  |  | . |  | . |  | . |  | . |  | . |  | . |  |
| Note. Tau² Estimator: Restricted Maximum-Likelihood | | | | | | | | | | | | | |
|  | | | | | | | | | | | | | |
